# Unfolding the role of exercise in the management of sleep disorders

**DOI:** 10.1007/s00421-024-05556-6

**Published:** 2024-07-20

**Authors:** Christoforos D. Giannaki, Giorgos K. Sakkas, Georgios M. Hadjigeorgiou, Mauro Manconi, Panagiotis Bargiotas

**Affiliations:** 1 Department of Life Sciences, School of Life and Health Sciences, University of Nicosia, 46 Makedonitisas Avenue, 1700 Nicosia, Cyprus; 2 School of Physical Education, Sport Science and Dietetics, University of Thessaly, Trikala, Greece; 3 Department of Neurology, Medical School, University of Cyprus, Nicosia, Cyprus; 4 Sleep Medicine Unit, Regional Hospital of Lugano, Neurocenter of Southern Switzerland, Lugano, Switzerland; 5 Sleep and Motion Laboratory, Medical School, University of Cyprus, Nicosia, Cyprus

**Keywords:** Aerobic exercise, Insomnia, Resistance exercise, Restless legs syndrome, Sleep apnea

## Abstract

Sleep disorders are prevalent among the general population and even more in individuals suffering from chronic diseases. Recent data reveal promising effects of physical exercise as a non-pharmacological approach for improving sleep and managing various sleep disorders. However, more studies with proper design and methodology should be conducted in the future to obtain a clearer understanding of the subject. The role of exercise in preventing and improving sleep disorders is probably much higher than what is currently exploited. To fully exploit the potential benefit of physical activity on sleep disorders in the future, it is necessary to identify the relevant tools to assess sleep–wake disorders and establish specific exercise protocols tailored to different sleep disorders. The present manuscript aims to review the literature on the use of exercise in managing selected sleep disorders. Regular exercise, including short-term aerobic activity, resistance training, and mind–body exercises, can effectively improve sleep quality, particularly in cases of insomnia and sleep-disordered breathing. Additionally, increasing evidence supports the effectiveness of aerobic and strength training, and body-mind exercises such as yoga in managing sleep-related movement disorders. Exercise can be a safe, affordable, and efficient tool in enhancing sleep quality and improving sleep disorders. Per se, regular exercise could play an adjuvant role alongside with established therapies, or a valid alternative when the pharmacological approach is limited by side effects, interactions, or inefficacy. More research is needed to define how exercise affects the physiology of sleep, and consequently how to use exercise in patients with sleep disorders.

**Graphical Abstract:**

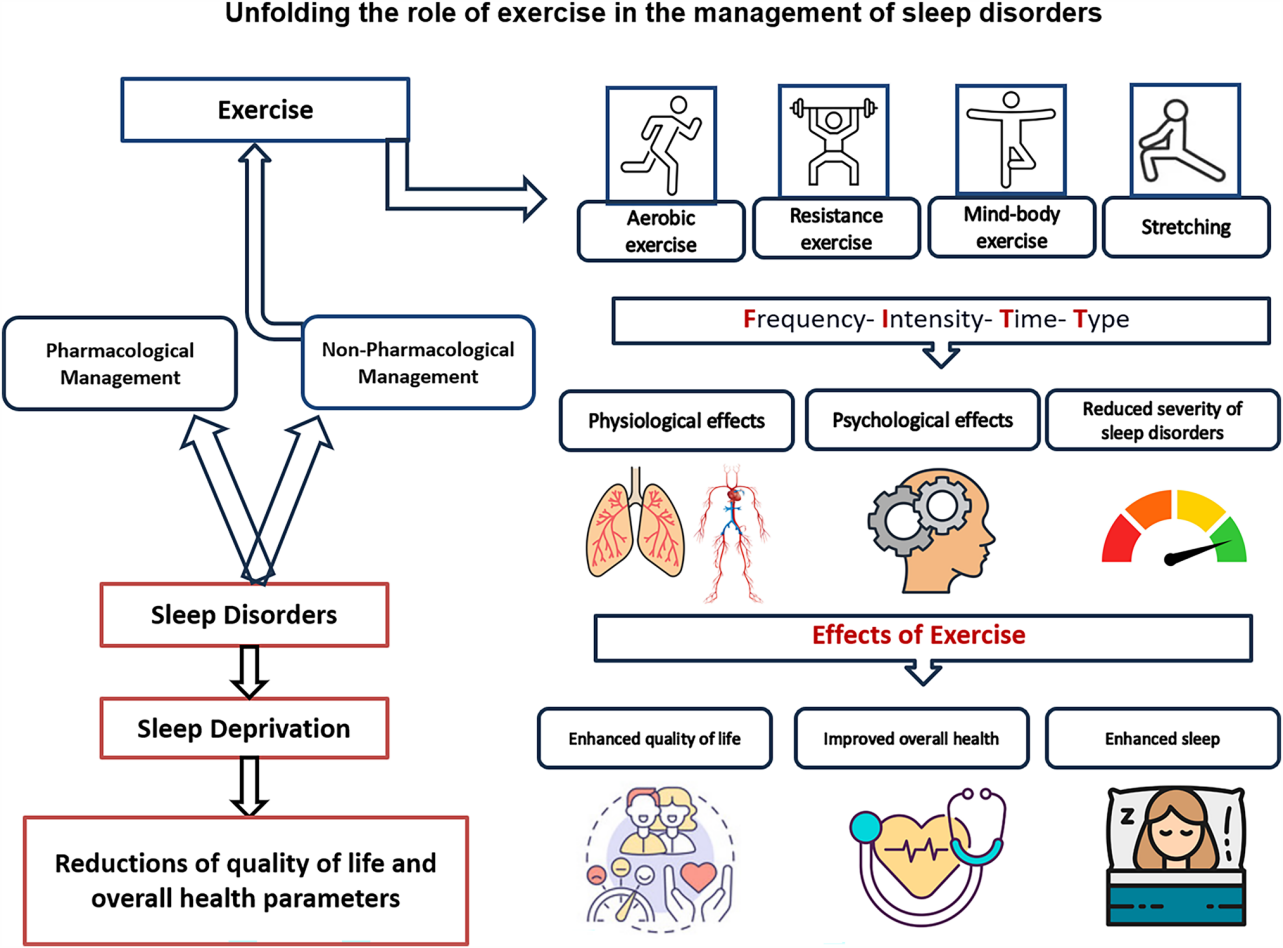

## Introduction

Sleep occupies one third of human life and plays a critical role in ensuring overall health and well-being across the lifespan. Accordingly, impaired and inadequate sleep leads to a cascade of detrimental effects on health and quality of life, with a negative impact on physical, mental, and social activities. Long-lasting sleep impairment and chronic untreated sleep disorders are well-established risk factors for high-impact enduring medical conditions like diabetes, high blood pressure, cardiovascular diseases, psychiatric illnesses and all-cause mortality (Cappuccio et al. 2010).

Sleep disorders and inadequate sleep are highly prevalent in the general population, therefore, it is not surprising that a great deal of research focuses on sleep and the consequences of sleep impairment. Approximately one-third of the population experience sleep-related disturbances (Chattu et al. 2018), and poor sleep stands as a primary health problem worldwide (Stranges et al. 2012). According to the literature, sleep disorders and poor sleep are associated with adverse effects on physiological, metabolic (Giannaki et al. 2014) and psychological parameters (Stranges et al. 2012), occupational performance and work-related accidents, social functioning, vigor (Partinen et al. 2021), cognitive function (Wang et al. 2022), and all-cause of mortality and morbidity (Yin et al. 2017; Cappuccio et al. 2010).

Poor sleep has a particularly strong impact on vulnerable individuals, especially those affected by other chronic diseases, further impairing mental and physical functioning, quality of life (Basnet et al. 2016), and their ability to perform normally in daily activities (Giannaki et al. 2014, 2018). Sleep loss can have detrimental consequences on body composition, particularly on musculoskeletal system. It can impair the synthesis of proteins within the myofibrils and sarcoplasm, with health and physical functioning consequences (Morrison et al. 2022). Moreover, sleep disorders have a very high economic burden on the healthcare systems and societies (Daley et al. 2009) not only due to the effect on health but also as a result of driving or work-related accidents (Uehli et al. 2014).

Treatment and prevention of sleep disorders require a combination of pharmacological and non-pharmacological interventions like behavioral lifestyle changes. Patients experiencing sleep symptoms should be guided towards embracing lifestyle modifications (i.e. diet and exercise for weight loss) as a preliminary approach before or in parallel to prescribed medications. Among the non-pharmacological approaches, it is well established that exercise training could induce favorable effects on sleep and, consequently, at multisystem level, on several health-related parameters. There is a growing amount of evidence supporting exercise training as a safe and effective intervention in preventing and improving the management of many chronic conditions, including diabetes, obesity, metabolic syndrome and cardiovascular diseases (Pedersen and Saltin 2015). Besides, systematic exercise training reflects positively on various physiological dimensions of the human body, providing substantial health-related benefits in terms of psychological, mental, social, and cognitive well-being, thereby enhancing the overall quality of life (Pedersen and Saltin 2015).

The high burden of sleep disorders in general population (Ferrie et al. 2011; Simonelli et al. 2018) and in particularly on patients with chronic diseases (Giannaki et al. 2014), has grown further during the COVID-19 pandemic (Partinen et al. 2021). The most common sleep disorders are insomnia, sleep-disordered breathing such as sleep apnea, restless legs syndrome and periodic limb movements, excessive daytime sleepiness and hypersomnia, circadian disorders and parasomnia. Considerable progress in diagnosing and treating sleep disorders has been made in the last decades, reflecting the growing interest of public health and research in sleep medicine. In particular, recent evidence proved the benefits of non-pharmacological approaches such as exercise on sleep disorders' symptoms and their consequences.

The current manuscript aimed to review the literature on the role of exercise in the management of the symptoms of selected sleep disorders. In particular, we focused on the advantages of exercise for every type of sleep disorder, speculating on the possible mechanism through which the exercise exerts its effects in relation to the specific disorder's pathophysiology.

## Exercise types and their intensity grading

Exercise can be broadly categorized into various types, including aerobic (i.e., running, swimming, and cycling), resistance (i.e., weightlifting and bodyweight exercises), and mind–body exercises (i.e., Yoga, Pilates and Tai Chi). It should be noted that each form of exercise possesses its own unique characteristics, and the adaptations and benefits associated with each type vary. For instance, aerobic exercise is highly effective in improving aerobic capacity and also aids in reducing body weight and fat (Weston et al. 2014). On the other hand, resistance training is particularly effective for enhancing strength and may also increase muscle mass (Marques et al. 2023). Conversely, mind–body exercises such as Yoga and Tai Chi can help reduce stress and increase range of motion (Li and Goldsmith 2012). Additionally, combining aerobic and resistance training into a single exercise regimen, often referred to as concurrent training, has been shown to provide comprehensive health benefits. This form of training harnesses the cardiovascular benefits of aerobic exercises alongside the muscle-strengthening benefits of resistance activities, potentially offering a balanced approach to physical fitness (Wilson et al. 2012).

To design and oversee effective and safe exercise programs, exercise professionals employ the F.I.T.T. principles, which stand for Frequency, Intensity, Time, and Type of exercise. These guidelines help in structuring exercise regimens that meet individual fitness goals while ensuring safety and sustainability (Garber et al. 2011). The intensity of these exercise forms is typically graded as low, moderate, or high. Low-intensity exercise, characterized by a lower exertion level, typically does not significantly elevate heart rate or breathing and can be sustained for extended periods. The primary benefits of low-intensity exercise include improved joint mobility, enhanced circulation, and stress reduction, making it particularly suitable for beginners, older adults, or those recovering from injury (Garber et al. 2011). Moderate-intensity exercises are generally sustainable and cause noticeable increases in heart rate and breathing (Garber et al. 2011). In contrast, high-intensity exercises, such as High-Intensity Interval Training (HIIT), involve short bursts of extreme activity alternated with intervals of lighter activity or rest, pushing the body to its metabolic limits (Foster et al. 2015).

## Sleep assessment methods

There are several ways to evaluate sleep, objective and subjective methods, and each method possesses its pros and cons aspects. Polysomnography (PSG) is considered the gold standard in defining sleep structure and consists in a multi-parameters monitoring employed to gather physiological data during sleep. PSG incorporates the use of electroencephalogram, electro-oculogram, electromyogram, electrocardiogram, pulse oximetry, airflow, and respiratory effort to concurrently and continuously capture neurophysiologic, cardiopulmonary, and various other physiological parameters throughout an extended period, typically spanning an entire night (overnight PSG) (Jafari and Mohsenin 2010; Markun and Sampat 2020). PSG offers valuable insights into the physiological alterations occurring in various organ systems in relation to different sleep stages and wakefulness. It allows both qualitative and quantitative documentations of abnormalities in sleep and wakefulness, the transitions between these states, and the physiological functioning of other organ systems influenced by the sleep process (Markun and Sampat 2020; Jafari and Mohsenin 2010).

Recent advancements in technology have significantly enhanced the performance of home-based PSG through the integration of portable devices. These innovative solutions have revolutionized the field of sleep monitoring by providing a less costly and reliable alternative to traditional in-laboratory video-polysomnography. Portable PSG devices leverage cutting-edge sensors and miniaturized components to capture comprehensive sleep data in the comfort of one’s home. The integration of wireless connectivity allows real-time transmission of valuable information to healthcare professionals, enabling remote monitoring and timely interventions. This technological progress enhances the overall accessibility of sleep diagnostics, making it more convenient for individuals to monitor their sleep patterns (a person's routine of going to bed, waking up, and napping behavior) and contribute to the advancement of sleep medicine (Withers et al. 2022). Notably, despite their practicality compared to conventional PSG, only a few portable PSG devices have been validated and proven accurate. Therefore, careful consideration is required when selecting a device for home-based PSG studies.

Actigraphy is a non-invasive method for monitoring motor activity and for the evaluation of patients with suspected sleep disorders over medium-long periods, making it a valuable tool in sleep medicine and research. This wrist-worn device utilizes accelerometers to record multidirectional movements, translating it into detailed information about an individual's daily motor activities and sleep–wake patterns. Actigraphy offers an easy and unobtrusive method of data collection, eliminating the need for cumbersome wires or intrusive sensors and excels in long-term data collection, allowing for continuous monitoring over extended periods (Patterson et al. 2023; Vlahoyiannis et al. 2020).

Despite some debate about its accuracy in assessing sleep-related parameters in patients with chronic insomnia disorder (Choi et al. 2017), actigraphy is commonly used for evaluating insomnia and circadian sleep–wake disorders. An additional valuable tool for assessing sleep hygiene, sleep–wake patterns, and subjective sleep quality features is the sleep–wake diary. By systematically recording sleep habits, wake times, and daily activities, it provides detailed insights into an individual's sleep routine (Carney et al. 2012). Actigraphy complements the sleep–wake diary by objectively measuring movement and activity levels, offering a comprehensive picture of diurnal and nocturnal behavior. Together, these methods can identify patterns and inconsistencies, guide personalized interventions, and track improvements over time, making them essential for both clinical assessment and self-monitoring of sleep hygiene and quality (Carney et al. 2012).

Finally, the field of sleep medicine benefits from a substantial array of validated and reliable questionnaires designed for the assessment of sleep–wake symptoms, including the Epworth Sleepiness scale, the Athens Insomnia Scale, the Fatigue Severity Scale, the Berlin Questionnaire for sleep apnea screening and others. Among these tools, the Pittsburgh Sleep Quality Index (PSQI) stands out as a particularly noteworthy instrument to assess overall sleep quality (a person’s satisfaction with their sleep, encompassing sleep initiation, maintenance, duration, and refreshment upon waking). PSQI is a widely used and well-established questionnaire comprising 19 self-reported items. Recognized for its reliability and validity, the PSQI provides a comprehensive and standardized measure of sleep quality over a 1-month period and serves as a valuable instrument in both clinical and research settings, facilitating the assessment of various aspects of sleep and aiding in the identification and follow-up of sleep disorders (Buysse et al. 1989; Vlahoyiannis et al. 2020).

## Exercise in sleep disorders

### Insomnia

Insomnia is the most common sleep disorder. Research has shown the presence of insomnia in 10–30% of the population globally, some even as high as 50% (Chattu et al. 2018). Insomnia is defined as difficulty getting to sleep or maintaining sleep or waking up too early, in association with daytime impairment in patients with adequate opportunity to sleep. According to the current diagnostic criteria, it can be classified in acute and chronic insomnia with 3 months as watershed (Sateia 2014).

Interesting data are nowadays available on the effect of physical activity on sleep parameters related to insomnia. Regular exercise leads to increased total sleep time, improved sleep quality and sleep architecture (Kubitz et al. 1996; Chennaoui et al. 2015). In addition, studies demonstrated that the beneficial effects of exercise on sleep is extended to patients suffering from insomnia (Xie et al. 2021). A recent meta-analysis on randomized controlled trials (RCTs) showed that regular exercise (physical or mind–body forms) improves sleep quality, daytime sleepiness, and insomnia symptoms (Xie et al. 2021). Interestingly, short-term (< 3 months) exercise was reported to be more effective on sleep quality compared with the long-term one (> 3 months). However, most studies did not include validated objective methods to assess sleep while many studies did not refer to patients with diagnosed insomnia (Xie et al. 2021). Another recent network meta-analysis of RCTs on patients with insomnia, illustrated that exercise was effective in ameliorating self-reported sleep onset latency (the duration between turning off the lights at bedtime and actually falling asleep) with small to medium effects (Baglioni et al. 2020).

Among the different exercise interventions, aerobic activity resulted as the most commonly used and effective one in improving insomnia symptoms and sleep quality. Aerobic exercise training can be carried out by employing a range of different levels of intensity. The conventional aerobic exercise activity of continuous moderate-intensity training effectively reduces insomnia symptoms (Xie et al. 2021). In recent years, high-intensity interval training (HIIT) has emerged as a very widely used exercise method. Initially, anecdotal reports raised doubts about the potential negative impact of HIIT on sleep, particularly when performed in the late evening. However, recent evidence suggests that these exercise regimens do not have a detrimental effect on sleep, at least for healthy individuals without insomnia (Vlahoyiannis et al. 2021; Thomas et al. 2020). However, future studies should investigate the impact of high-intensity exercise when conducted in the late evening in patients with insomnia.

Mind–body exercises such as Yoga and Tai-Chi are also effective in patients with insomnia, probably by favoring relaxation, thereby assisting in improving sleep quality and duration-sleep initiation (Xie et al. 2021). A recent systematic review and meta-analysis on the effects of Yoga on insomnia symptoms in women with sleep problems showed a significant positive effect on subjective sleep quality (Wang et al. 2020). The same meta-analysis also showed a positive association between the duration of Yoga classes and the improvement of sleep quality and other health-related outcomes.

There is evidence that even a single bout or a longer period of resistance training might improve sleep quality. Passos and colleagues examined the effects of acute aerobic exercise at different intensities over moderate-intensity strength training in patients with chronic insomnia (Passos et al. 2010). Aerobic exercise of moderate intensity (performed at an intensity that corresponded to the first ventilatory threshold) was the most effective form of exercise in terms of sleep improvement (55% reduction in sleep onset latency; 18% increase in total sleep time, and 13% increase in sleep efficiency as assessed by PSG) and anxiety reduction. Chronic exercise can also be beneficial for patients with insomnia. Four months of resistance exercise training (50% of 1-Repetition Maximum (RM) in the first month and 60% of 1-RM in the remaining three months) reduced the insomnia severity index scale score by 10.5, while in parallel, reduced sleep latency by 19.6 min (assessed by actigraphy) and improved sleep efficiency by 4.4% (D'Aurea et al. 2019).

Exercise might exert its effectiveness on insomnia through several pathways. Regular aerobic exercise is linked to enhanced general well-being, encompassing cardiovascular health, metabolic efficiency, and weight control (Driver and Taylor 2000; Chennaoui et al. 2015). Moreover, physical activity lead to physical fatigue, thus facilitating sleep (Driver and Taylor 2000). Regular aerobic exercise has been shown to reduce symptoms of anxiety and depression (Giannaki et al. 2013a), which are often in comorbidity with chronic insomnia (Staner 2003). Furthermore, exercise modulates sleep-related hormone secretion, increasing melatonin and reducing stress-activating hormones such as cortisol (Al-Sharman et al. 2019; Driver and Taylor 2000) and insulin resistance (Chennaoui et al. 2015).

Resistance exercise can produce weight loss, which in turn may help in improving sleep. Additionally, this form of exercise promotes the secretion of growth hormone and reduces cortisol levels, which contribute to better sleep patterns.

Finally, mind–body exercises ameliorate insomnia symptoms (Xie et al. 2021), probably by reducing stress and anxiety, enhancing regular breathing, and relieving muscle tension (Woodyard 2011).

### Sleep disordered breathing

#### Obstructive sleep apnea

Sleep apnea is a condition characterized by recurring interruptions of breathing while sleeping, which can be attributed to either the obstruction of the upper airways (obstructive sleep apnea) or an impaired drive in the neurological regulation of breathing (central sleep apnea) (Epstein et al. 2009).

Obstructive sleep apnea (OSA) is the most common sleep-disordered breathing. It is complex multi-faceted disease associated with a variety of symptoms and long-term mainly metabolic and cardiovascular complications (Epstein et al. 2009; Chang et al. 2023). Notably, recent epidemiological surveys estimated its prevalence as huge, with almost one billion people affected worldwide, with a positive relationship with age, male sex and overall obesity (Benjafield et al. 2019).

Patients with chronic diseases such as heart failure, kidney diseases and hypertension have greater fluid and fat accumulation in the neck, which is implicated with OSA and its severity (Andrade and Pedrosa 2016). OSA patients also show a reduced physical activity and poor functional capacity. According to a recent meta-analysis, individuals with OSA exhibit a notably lower average daily step count than those without the condition (Mendelson et al. 2018). Furthermore, the level of physical activity expressed by OSA patients falls significantly below the minimum recommended by the scientific community in order to enhance overall health and minimize the likelihood of developing pathological conditions and health-related issues (Mendelson et al. 2018).

Over the past few decades, the medical community has devoted significant attention to OSA owing to its strong connection with both health and quality of life. OSA can negatively affect physical performance and impair daytime functioning (Sakkas et al. 2008a), favoring fatigue, daytime sleepiness, and cardiovascular and metabolic diseases (Abbasi et al. 2021). The first-line treatment of OSA is represented by nocturnal ventilation with continuous positive airway pressure (CPAP), however, the long-term tolerability of CPAP remains an issue. Alternative treatments include oral appliances, surgical interventions, neuromodulation and weight loss (Patil et al. 2019).

Regular exercise has been demonstrated to be beneficial in alleviating the symptoms associated with sleep apnea (Iftikhar et al. 2014). In the meta-analysis from Iftikhar and colleagues, it was discovered that engaging in exercise training for 3–6 months can significantly decrease the apnea–hypopnea index (AHI) by approximately 32% in patients with OSA (Iftikhar et al. 2014). In a recent study by Lins-Filho and colleagues in patients with OSA, HIIT for 12 weeks effectively reduced OSA symptoms (AHI was reduced by 8.6 events per hour), total sleep time was increased by 51 min, sleep efficiency increased by 12% and cardiorespiratory fitness increased by 4.8 ml/kg/min. The HIIT regime was performed on a treadmill and consisted of 5 periods of 4 min of exercise at 90–95% of maximum heart rate (HRmax) interspersed with 3 min at 50–55% of HRmax. These findings indicate a promising role of high-intensity exercise as an effective and time-efficient method to face OSA. However, more research is needed to extract more solid conclusions regarding the effectiveness of HIIT programs on OSA symptoms (Lins-Filho et al. 2023).

**Fig. 1:**
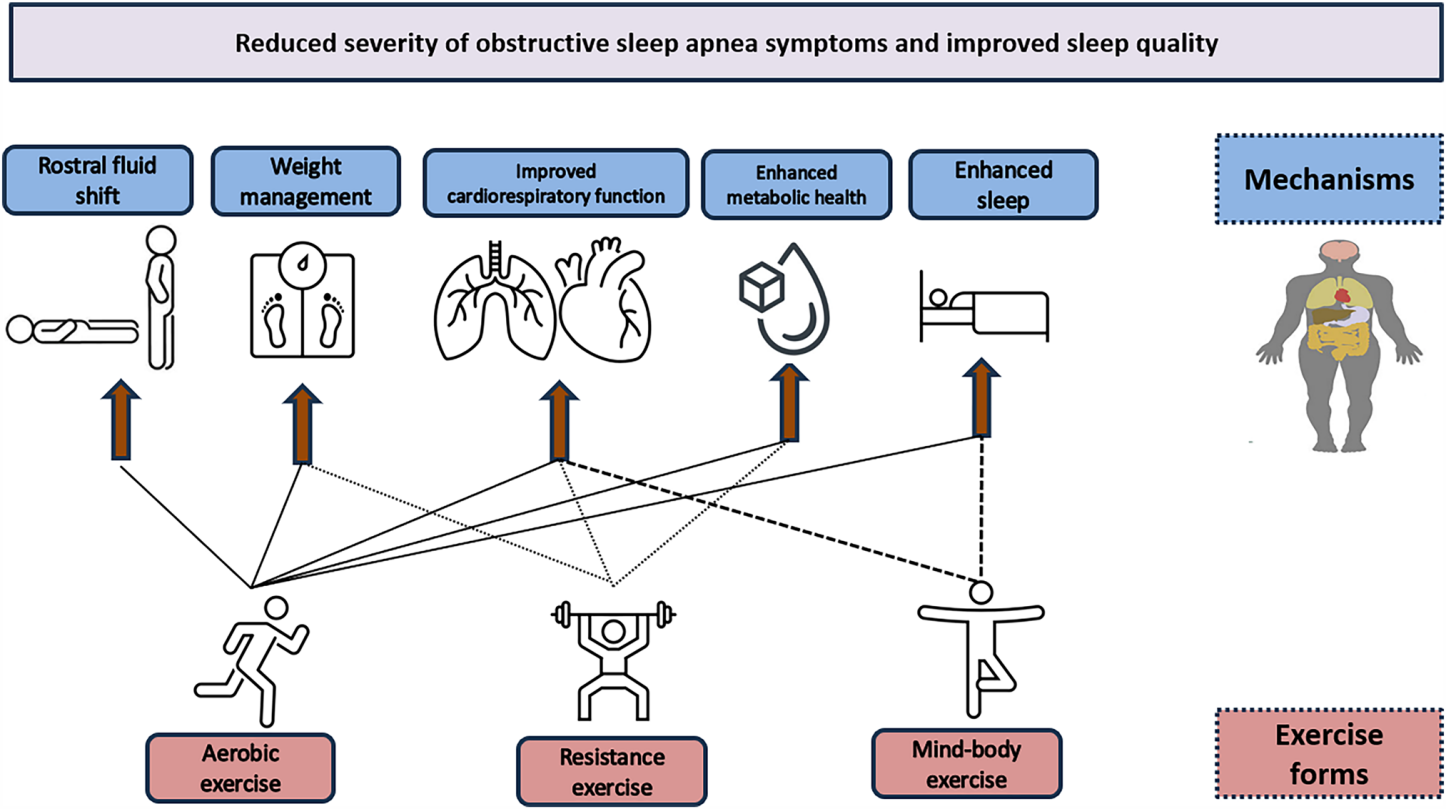
Potential mechanisms through which the different forms of exercise training can alleviate symptoms of sleep apnea and enhance the quality of sleep. Engaging in aerobic exercise training can help in weight reduction, enhanced cardiovascular and respiratory function, as well as improved metabolic health. Moreover, it can aid in reducing rostral fluid retention and promote better quality sleep. On the other hand, resistance exercise is also beneficial for weight control and can contribute to improved cardiovascular and metabolic health. Additionally, mind–body exercises like Yoga and Tai-Chi have been observed to positively impact sleep apnea severity primarily through their influence on overall sleep patterns and respiratory function

Exercise can relieve OSA and its symptoms in various ways. First, exercise training can ameliorate OSA through its positive effects in weight reduction (37). Excessive body weight is one of the most important risk factors for OSA, while there is an inverse relationship between weight loss and severity of OSA. Indeed, research findings indicate that a decrease of 10% in the body mass index (BMI) is related to a notable decrease of 30% in the AHI (Newman et al. 2005). However, recent systematic reviews highlight the fact that exercise training, and especially the combination of aerobic and resistance exercise, are effective in reducing OSA, independently of losing weight (Kline et al. 2011; Sengul et al. 2011). For instance, Kline and colleagues, combining aerobic and resistance exercise training for a 12 weeks period were able to reduce AHI by 23%, and the oxygen desaturation index, without affecting body weight (Kline et al. 2011). Second, exercise can result in improved cardiovascular health and respiratory function, enhancing sleep stability (i.e. regularity in sleep duration, timing, and quality) and respiration (Mendelson et al. 2018). Exercise can reduce rostral fluid retention, favoring fluid redistribution, while its effect on cardiovascular system and weight management can regulate fluid balance in the body and potentially reduce the severity of rostral fluid accumulation (Kline 2016). Moreover, it is well known that exercise (aerobic and resistance) can enhance insulin sensitivity and glucose metabolism, both associated with impaired sleep, cardiovascular and cardiometabolic diseases (Briancon-Marjollet et al. 2015) (Fig. 1). Specific exercises (i.e. such that are included in Yoga and Tai-Chi programs) that target proper posture and alignment of the body can stabilize upper airways, promote respiratory function while sleeping (Ratarasarn and Kundu 2020) and reduce stress and anxiety which are often present in OSA patients (Wong et al. 2021). Furthermore, engaging in systematic exercise training offers notable anti-inflammatory benefits that could potentially alleviate certain underlying factors associated with OSA.

#### Central sleep apnea and Cheyne-Strokes respiration

Central sleep apnea (CSA) is characterized by an impairment of the respiratory drive during sleep, leading to repetitive pauses of breathing and eventually to insufficient or absent ventilation and consequently compromised gas exchange (Eckert et al. 2007). While the precise pathophysiology of CSA is not entirely elucidated, several mechanisms have been proposed. These include instability in the ventilatory control system from augmented chemosensitivity to carbon dioxide, and fluctuations in pulmonary blood flow (Bradley and Floras 2003). Literature on the impact of exercise on CSA is limited. Aerobic exercise has been reported to improve CSA symptoms severity. In the study by Yamamoto et al. in chronic heart failure patients with CSA, six months of aerobic exercise training effectively reduced the severity of sleep apnea (AHI was significantly decreased from 24.9 to 8.8 and the numbers of central sleep apnea per night was significantly decreased from 152 to 50, respectively) and improved exercise capacity (Yamamoto et al. 2007). Suggested mechanisms for the beneficial impact of exercise on CSA include improving autonomic balance, reducing endothelial dysfunction, increasing central chemosensitivity, potentially as a result of elevated catecholamine levels during exercise, and the synergy of these factors (Hambrecht et al. 1998; Tomita et al. 2003). These mechanisms may collectively contribute to stabilize the ventilatory drive, ultimately ameliorating central sleep apnea. To the best of our knowledge, there is no research specifically focused on the effects of resistance exercise or other forms of exercise, such as mind–body exercise on CSA. Future studies should investigate the effects of systematic exercise training of different forms, durations and intensities in patients with CSA.

Cheyne–Stokes respiration (CSR) is often categorized as a specific type of CSA and is characterized by periodic breathing with cyclic variations in the depth of breaths, alternating between hyperventilation and apnea. This breathing pattern is predominantly observed in patients during sleep, but it may also manifest in awake individuals. CSR is commonly linked with serious health conditions such as heart failure, stroke, and traumatic brain injuries, suggesting significant underlying medical issues (Javaheri and Dempsey 2013). The pathophysiological mechanisms of CSR include heightened sensitivity to carbon dioxide and a delayed response in the respiratory control system, leading to overcompensation and irregular breathing patterns (Lanfranchi and Somers 2003). Currently, there appears to be no research specifically investigating the effects of exercise on patients with Cheyne–Stokes respiration.

### Restless legs syndrome

RLS (also known as Willis–Ekbom disease), is a sensory-motor neurological disorder characterized by an overwhelming urge to move mainly legs, usually accompanied with unpleasant sensations. The symptoms begin or worsen during rest and inactivity periods, mainly at night, whereas temporary improve by movement (Allen et al. 2003; Allen et al. 2014). Approximately 80% of the RLS patients show periodic limb movements during sleep (PLMS) (Montplaisir et al. 1997). This common feature of RLS, consists in stereotyped repetitive foot and knee flexion occurring mainly during non-rapid eye movement (NREM) sleep, associated with potential disrupting cortical and autonomic arousals and motor restlessness (Allen et al. 2014). PLMS could be recognized and scored by means of an overnight polysomnography (Allen et al. 2014).

RLS can typically be distinguished in primary (idiopathic RLS) or RLS-associated with major diseases (symptomatic RLS) such as End-Stage Renal Disease (ESRD), iron deficiency, pregnancy, drug related and others) (Trenkwalder et al. 2016). The prevalence of RLS in the general population ranged from 3 to 12% (Innes et al. 2011), with 1.5–2.7% of the cases considered clinically significant thus worthy to be treated (Silber et al. 2021). In pathological conditions implicated in symptomatic forms of RLS, the prevalence of RLS is even higher. For instance, the prevalence of RLS in ESRD patients (also known as uremic RLS) reaches approximately 30% of the population (Giannaki et al. 2014). Besides this, symptomatic forms of RLS are associated with a worsened severity of symptoms and lower quality of life compared to idiopathic RLS (Gkizlis et al. 2012), probably due to the combined negative effect of sleep disorder and medical condition. The pathophysiology of RLS is still unclear, with genetic predisposition and external factors like iron deficiency, playing a possible combined role in altering dopaminergic and glutamatergic neurotransmission (Khachatryan et al. 2022).

It is well known that RLS impact the quality and duration of sleep, impacting quality of life (Giannaki et al. 2014). Evidence suggests a negative effect of RLS disorders on musculoskeletal health and function. For instance, in the case of RLS patients with multiple sclerosis, evidence reveals that the patients with RLS may experience further reduction in functional capacity compared to their RLS-free counterparts (Giannaki et al. 2018), with the severity of RLS associated with reduced functional capacity (Cederberg et al. 2022). Muscle characteristics and mass appeared to be also worsen in RLS patients, with increased muscle atrophy to be presented in uremic patients (Giannaki et al. 2011a) and significant remodeling in capillary geometry in idiopathic RLS patients (Larsson et al. 2007). RLS can lead to sleep loss, which is linked to hormonal shifts that favor catabolism such as increased cortisol and decreased testosterone secretion, while it hampers the process of muscle protein synthesis (Morrison et al. 2022).

Management of RLS symptoms include both pharmacological and non-pharmacological approaches. The first line pharmacological treatment for RLS is represented by alpha-2 delta ligands and dopamine agonists (Garcia-Borreguero et al. 2013). Other drug approaches include opioids, intravenous iron infusion, and anti-glutamatergic drugs (Garcia-Borreguero et al. 2013).

On the other hand, recent studies conducted in both idiopathic and symptomatic RLS reported significant reduction of RLS symptoms induced by acute or chronic exercise training. Aukerman et al. were the first to examine the effectiveness of exercise training in idiopathic RLS (Aukerman et al. 2006). A combination of aerobic and strength exercise training, performed three times per week, was successful in reducing the severity of RLS measured by the standard international RLS rating scale by approximately 40%. The aerobic part consisted in walking for 30 min at an intensity of 40–60% of the patients’ age-predicted maximum heart rate and with a rating of perceived exertion scale of 10 to 13, indicating a moderate intensity. The exercise prescription for strength exercise training was set at 50% of the estimated 1-Repetition Maximum (1-RM). The beneficial effect of aerobic exercise in patients with idiopathic RLS was confirmed by more recent studies (Coban et al. 2023).

Sakkas and colleagues first examined the effects of both acute and chronic exercise training in uremic symptomatic RLS. Intradialytic aerobic exercise (cycling during the hemodialysis session) was effective in reducing RLS symptoms by 42 to 58% (Giannaki et al. 2013b, 2013a; Sakkas et al. 2008b), and improving sleep quality by 20 to 50%, functional capacity, quality of life and depression. Herein, the intensity of aerobic exercise was moderate (60–65% of the patients’ maximal exercise capacity) for a duration of 45 min, performed three times per week (Sakkas et al. 2008b; Giannaki et al. 2013b, 2013a). Notably, PSG was not used in the above studies, therefore, no evidence exists regarding the effect of aerobic exercise in sleep architecture and PLMS in patients with uremic RLS. In general, the effectiveness of exercise training in reducing RLS symptoms in uremic patients is relatively well-examined compared to other forms of RLS (idiopathic and symptomatic) (Song et al. 2018). Aerobic exercise (using an arm cycle ergometer) has been shown to reduce the severity of PLMS in patients with spinal cord injury (De Mello et al. 2002).

A critical issue concerning exercise and RLS regards the intensity of the exercise intervention. Anecdotal evidence indicates that individuals with RLS should avoid engaging in vigorous and strenuous forms of exercise and physical activities, as these may worsen RLS symptoms. It was unclear whether the observed improvement in the severity of RLS among uremic patients was due to exercise-induced systemic effects or merely to acute relief from movement-induced RLS. To test the above hypothesis, Giannaki and colleagues conducted a six-month randomized control trial by using intradialytic exercise of moderate intensity versus sham-exercise (no resistance) in uremic RLS (Giannaki et al. 2013a). Patients underwent to progressive exercise reported a reduction of RLS severity by 58%, whereas a non-significant reduction of 17% was observed in the sham-exercise group (Giannaki et al. 2013a). In line with these findings, Cederberg et al. examined whether acute aerobic exercise of different intensities can affect the severity of RLS symptoms in the night of the exercise session as well as 24 and 48 h post exercise (Cederberg et al. 2018). This study was conducted in idiopathic RLS patients and it showed that that acute exercise, at either 50% or 70% of heart rate reserve, may have an immediate positive effect on RLS symptoms that dissipate within 48 h after exercise.

Motor restlessness often appear during the hemodialysis session, when the patients are requested to be rest in sitting or lying position for hours. Acute intradialytic cycling was also effective in reducing legs movements by approximately 30% during hemodialysis in uremic RLS patients (Giannaki et al. 2010).

It seems that also mind–body forms of exercise training have a benefit on symptoms of RLS. Yoga exercise for eight weeks (Innes et al. 2013) or 12 weeks (Innes et al. 2020) appeared to be effective in terms of reducing the RLS symptoms in idiopathic RLS. Similarly, stretching exercise of lower extremities for 8 weeks can relief RLS symptoms in hemodialysis patients (Aliasgharpour et al. 2016). Both of the above mentioned studies showed that these forms of exercise can reduce RLS severity by 35–50% as assessed by the IRLS severity scale.

**Fig. 2:**
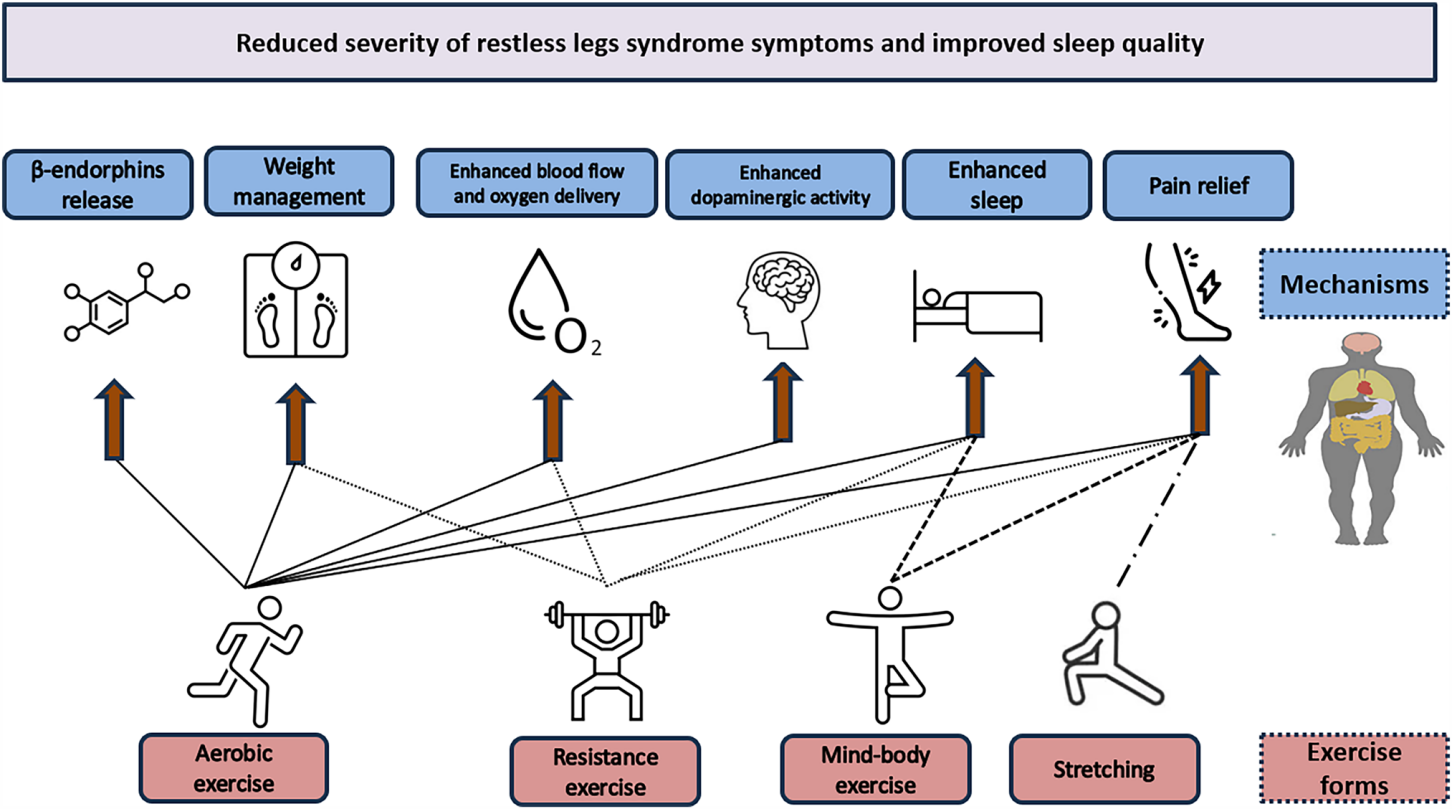
Hypothetical mechanisms through which diverse types of exercise training can ameliorate symptoms of restless legs syndrome and enhance the quality of sleep. Engaging in aerobic exercise can promote the secretion of β-endorphins, thereby diminishing sensations of pain. Additionally both aerobic and resistance exercise stimulate the activity of dopamine in the brain, and have the potential to facilitate weight loss, enhance blood circulation, increase oxygen supply to the body and the brain, and augment both the quality and duration of sleep. Mind–body forms of exercise can relief the severity of restless legs syndrome and improve sleep. Stretching exercise can help also in the severity of restless legs syndrome, mainly through its pain relief properties

The exact mechanism by which exercise could induce amelioration of RLS symptoms is still unknown. RLS is a complex sensory and motor disorder, and each form of exercise training can influence different adaptive aspects which can theoretically help on the reduction of the severity of RLS symptoms. For instance, regarding aerobic exercise, evidences report an increased release of endogenous opioids (pain-relieving substances) such as β-endorphins after exercise training (Esteves et al. 2009). Furthermore, it has been observed that the rise in beta-endorphin levels directly corresponds with the increase in overall β-endorphins levels (Esteves et al. 2009). In the case of uremic RLS, previous studies observed significant improvements in dialysis adequacy following aerobic intradialytic exercise (Giannaki et al. 2011b) and theoretically, this could be also a mechanism that can explain the amelioration of RLS symptoms with exercise training. Other possible explanations about the aerobic exercise effects on reducing RLS severity are its beneficial effects on cardiovascular function (i.e. increased blood flow) and oxygen delivery system. There are evidences that RLS severity might be positively related with peripheral hypoxia, implying that impaired oxygen delivery to the periphery may play a role in the pathogenesis of the syndrome (Salminen et al. 2014). Additionally, engaging in physical activity is widely recognized for its ability to enhance the overall perception of sleep quality and positively impact on various psychological aspects (Fig. 2). The diagnosis of RLS, as well the evaluation of its severity, relay on subjective reports by the patient (Allen et al. 2014). Given that physical activity can improve in general patient's overall well-being and quality of sleep, it is possible that this reflects in different and better subjective perception of the syndrome's severity.

Similarly, it is still a matter of discussion on how body–mind exercises such as Yoga can reduce RLS severity. A possible explanation is that these forms of exercise reduce the sensory symptoms and pain while improving sleep. Indeed, in the study of Innes et al. the patients also showed significant improvements in sleep, perceived stress, and mood along with the reduction of RLS severity (Innes et al. 2013). Finally, in a recent study by Coban and co-workers, stretching exercise was equally effective as aerobic exercise in reducing symptoms in idiopathic RLS patients (Coban et al. 2023).

It is well known that the pharmacological treatment of RLS with dopamine agonists (especially with levodopa) may induce a long- term paradoxical complication named “augmentation”. It appears mainly with relatively high dose of dopamine-agonists and consists on a paradoxical spreading of symptoms in other body parts further than legs, an anticipation of symptoms appearing across the day and a worsening of symptoms by increasing the dose of the drug. A common finding of all published studies is that both physical and mental exercises are free from the augmentation phenomenon. Therefore, exercise training can be potentially useful also in such groups of patients at risk or in a state of augmentation or even for patients with mild RLS.

## Future research directions

1. Future studies based on physical exercise should use objective gold standard methodology (i.e. polysomnography-PSG) to assess sleep. This has been partially done only in the field of sleep apnea syndromes. However, it is common for studies exploring the effects of exercise training on other sleep disorders to neglect the utilization of PSG.
2. There is a need for tailored exercise programs with longer duration, using different intensities and larger sample size (i.e. in studies with uremic RLS patients) in order to extract solid conclusions. The description of exercise protocols, which includes specific details about the exercise training program based on the FIIT (Frequency, Intensity, Time, and Type) principle, is often inadequately articulated.
3. Development of specific pre-sleep training programs dedicated to the management of different sleep disorders could also be a target for future studies. Moreover, in recent times, numerous research studies have delved into the impacts of different meals consumed before sleep on various aspects related to sleep in healthy individuals, yielding positive outcomes (Vlahoyiannis et al. 2018; Binks et al. 2020). In the future, it would be worthwhile for further studies to explore the possibility of integrating pre-sleep meal plans with specialized pre-nocturnal training programs for individuals suffering from sleep disorders.
4. There is a scarcity of studies that delve into the physiological mechanisms underlying the potential of exercise to alleviate the severity of sleep disorders, particularly in the case of RLS. Consequently, instead of relying on robust evidence, numerous assumptions are made regarding the ability of exercise to diminish the severity and consequences of sleep disorders.
5. No evidence exist regarding the effects of exercise training on the severity of rapid eye movement behavior disorder and central hypersomnolence disorder (CHD). Both disorders should be the target for future RCTs.

## Conclusion

Exercise training of various type and entity seems to be effective with regard to the amelioration of the severity of sleep disorders, improving in parallel the patients’ functionality and many aspects of quality of life. Future research is needed to investigate the optimal exercise prescription of the management of sleep disorders and unfold the physiological mechanisms by which exercise relief symptoms severity.

## Data Availability

Data sharing is not applicable to this article as no datasets were generated or analyzed during the current study.

## Abbreviations

AHI: Apnea–hypopnea index
BMI: Body mass index
CPAP: Continuous positive airway pressure
CSA: Central sleep apnea
CSR: Cheyne–Strokes respiration
ESRD: End-stage renal disease
HIIT: High-intensity interval training
HRmax: Maximum heart rate
NREM: Non rapid eye movement
OSA: Obstructive sleep apnea
PLMS: Periodic limb movements during sleep
PSG: Polysomnography
RCT: Randomized controlled trial
RLS: Restless legs syndrome
RPE: Rating of perceived exertion
1-RM: 1-Repetition maximum
